# Tinea Cruris

**Published:** 1910-10-08

**Authors:** 


### TINEA CRURIS.

Tinea cruris, or ringworm of the inguinal, scrotal, and perineal regions, may be due to different parasites in different cases, all belonging to the genus known as epidermophyton. The two best known are the *Epidermophyton Castellanii*, whose colonies in maltose agar are whitish and later develop a greenish colour; and the *Epidermophyton Perneti*, whose colonies in maltose agar are of a delicate pinkish colour in primary cultivations, the tint disappearing in subcultures. A third species has been more recently discovered by Castellani, whose cultural characterstics are as follows: On Sabouraud's agar the growth begins to appear four to six days after inoculation as a raised red spot, which gradually enlarges. The full-growth colonies are of a deep red colour, either with a central knob or crateriform, and they are partly covered, especially the central knob, by a white *duvet*. In old cultures the white *duvet* is larger and thicker and covers the whole growth, and may hide the red pigmentation almost completely. The pigmentation has not been lost in subcultures so far. On glucose agar the cultures are of a very deep blood-red colour, and a portion of the medium takes the same colour. In old cultures a large amount of white *duvet* is present all over the surface of the growth, but on scraping this out the red pigmentation is extremely well marked. On mannite the colonies are also deep red, covered with white *duvet*, with central knob. After a time the whole surface of the growth shows abundant whitish fluff.

In liq. potass. preparations from the affected parts mycelial tubes and free spores are observed, identical to what one sees in *Epid. cruris* and *Epid. Perneti*, and similar to any trichophyton of the megalosporon type. The spores are large, globular, 4 to 6 $\mu$ in diameter, with a double contour. The mycelial tubes, 2 to $3\frac{1}{2}$ $\mu$ in breadth, are straight or bent or variously shaped.

Owing to the deep red colour of the colonies in mannite the fungus has been termed the *Epidermophyton rubrum*.

